# Platelet Lipidome Fingerprint: New Assistance to Characterize Platelet Dysfunction in Obesity

**DOI:** 10.3390/ijms23158326

**Published:** 2022-07-28

**Authors:** Gaëtan Chicanne, Maria N. Barrachina, Anaelle Durbec, Justine Bertrand-Michel, Sara Troitiño, Lidia Hermida-Nogueira, Aurelio M. Sueiro, María Pardo, Bernard Payrastre, Ángel García

**Affiliations:** 1 Institute of Metabolic and Cardiovascular Disease, Inserm UMR1297, University of Toulouse 3, 31024 Toulouse, France; gaetan.chicanne@inserm.fr (G.C.); anaelle.dubrec@inserm.fr (A.D.); justine.bertrand-michel@inserm.fr (J.B.-M.); 2 Platelet Proteomics Group, Center for Research in Molecular Medicine and Chronic Diseases (CIMUS), Universidade de Santiago de Compostela and Instituto de Investigación Sanitaria de Santiago (IDIS), 15706 Santiago de Compostela, Spain; maria.barrachina@childrens.harvard.edu (M.N.B.); s.troitino@usc.es (S.T.); lidiahermidanogueira@gmail.com (L.H.-N.); 3 Vascular Biology Program, Boston Children’s Hospital, Harvard Medical School, Boston, MA 02115, USA; 4 MetaboHUB-MetaToul, National Infrastructure of Metabolomics and Fluxomics, 31077 Toulouse, France; 5 Grupo de Endocrinología Molecular y Celular, Instituto de Investigación Sanitaria de Santiago (IDIS), Servicio de Endocrinología, Xerencia de Xestión Integrada de Santiago (XXS), 15706 Santiago de Compostela, Spain; aurelio.manuel.martis.sueiro@sergas.es; 6 Grupo Obesidómica, Instituto de Investigación Sanitaria de Santiago (IDIS), Xerencia de Xestión Integrada de Santiago, 15706 Santiago de Compostela, Spain; maruxapardo@hotmail.com; 7 Laboratory of Haematology, University Hospital of Toulouse, 31059 Toulouse, France

**Keywords:** platelets, obesity, lipidomics

## Abstract

Obesity is associated with a pro-inflammatory and pro-thrombotic state that supports atherosclerosis progression and platelet hyper-reactivity. During the last decade, the platelet lipidome has been considered a treasure trove, as it is a source of biomarkers for preventing and treating different pathologies. The goal of the present study was to determine the lipid profile of platelets from non-diabetic, severely obese patients compared with their age- and sex-matched lean controls. Lipids from washed platelets were isolated and major phospholipids, sphingolipids and neutral lipids were analyzed either by gas chromatography or by liquid chromatography coupled to mass spectrometry. Despite a significant increase in obese patient’s plasma triglycerides, there were no significant differences in the levels of triglycerides in platelets among the two groups. In contrast, total platelet cholesterol was significantly decreased in the obese group. The profiling of phospholipids showed that phosphatidylcholine and phosphatidylethanolamine contents were significantly reduced in platelets from obese patients. On the other hand, no significant differences were found in the sphingomyelin and ceramide levels, although there was also a tendency for reduced levels in the obese group. The outline of the glycerophospholipid and sphingolipid molecular species (fatty-acyl profiles) was similar in the two groups. In summary, these lipidomics data indicate that platelets from obese patients have a unique lipid fingerprint that may guide further studies and provide mechanistic-driven perspectives related to the hyperactivate state of platelets in obesity.

## 1. Introduction

Obesity, defined as abnormal or excessive fat accumulation, constitutes a major health problem as it is associated with increased morbidity and mortality. According to the World Health Organization, adult obesity is a body mass index (BMI) greater than or equal to 30 kg/m^2^ [1]. Obesity is known to be a major risk factor for atherosclerotic cardiovascular disease (CVD) [2], where platelets play a pathogenic role in the development of heart attack and stroke [3]. Platelets are non-nucleated cell fragments released from their precursor cell, megakaryocytes, that play an essential role in primary hemostasis and in the pathophysiology of atherothrombosis [4]. Platelets are highly reactive to extracellular stimuli through the activation of a variety of specific membrane receptors for soluble agonists or adhesive proteins allowing platelet activation, adhesion, secretion and aggregation. It has been described that obese patients have increased platelet responsiveness and reduced sensitivity to antiplatelet agents, particularly when is associated with type 2 diabetes mellitus or with metabolic syndrome [3,5].

Green et al. reported that washed platelets from obese patients are hypo-reactive to thrombin stimulation and exhibit a deficiency in SERCA3-dependent adenosine diphosphate (ADP) secretion, and proposed that the hyper-responsiveness of platelets from obese patients is due to plasma components [6]. Recent studies also demonstrated that obese patients have an altered platelet transcriptome and increased platelet activation, which is partly attenuated by bariatric surgery [7]. In line with the latter, our group showed by using (phospho)proteomics that platelets from obese patients are altered in SFK (Src-family kinase)- and (hemi)ITAM (immunoreceptor tyrosine-based activation motif)-related signaling pathways and vesicle transport proteins, in addition to having increased surface expression of the collagen receptor GPVI [8,9], which correlates with a platelet hyperactivation state.

Although platelet function is predominantly regulated and altered by surface receptor and cytoskeletal organization, it is also reliant on lipid membrane composition and production of bioactive lipid mediators [10]. Upon activation, the lipid composition of the platelet membrane is modified, leading to the generation of lipid messengers accompanied by a redistribution of phospholipids between the two leaflets of the plasma membrane and allowing an increased binding of coagulation factors [10]. With technology advancements, lipidomic analyses have started to unravel the multifaceted aspects of the platelet lipidome, considering them a treasure trove of diagnostic and prognostic biomarkers for CVD [11].

Even though it has been observed that obesity-induced alterations in plasma lipid composition are associated with platelet activation [5], little has been studied about the role of lipids altering platelet reactivity in these types of patients. For these reasons, the main goal of this study was to evaluate the platelet lipidomic fingerprint in obesity. We used a targeted comparative lipidomic analysis to compare resting platelets from twelve non-diabetic, severely obese patients (BMI > 40 kg/m^2^) and their age- and gender-matched lean controls. This study was performed on a small cohort of patients, and the results may be a guide for further mechanistic studies to obtain deeper insights into the behavior of platelets in obesity.

## 2. Results

### 2.1. Clinical Characteristics of Patients

As indicated in Table 1 and Supplementary Table S1, twelve patients were included in the study with a diagnosis of severe obesity. All of them were non-diabetic, had a BMI > 40 kg/m^2^ and were 42.08 ± 11.09 years old at the time of inclusion. Compared to their age- and gender-matched lean controls, obese patients had significantly increased platelet and leukocyte counts, as expected. Notably, no significant differences were found regarding the mean platelet volume (MPV) and platelet distribution width (PDW), although the MPV levels were slightly higher in the obese group. Nevertheless, MPV data are in line with previous ones from similar cohorts we analyzed by proteomics [8].

Obesity is associated with an altered lipid plasma composition that leads to an increased cardiovascular risk [5]. According to the data, obese patients had significantly reduced levels of high-density lipoprotein (HDL) cholesterol and increased levels of triglycerides (TGs), as expected. The glycemia levels were significantly higher in the obese group compared to the lean controls (Table 1). Of note, one obese patient out of twelve was taking cholesterol-lowering drugs and skeletal muscle relaxants and two were receiving angiotensin-converting enzyme (ACE) inhibitors to treat hypertension.

### 2.2. Neutral Lipids: Free Cholesterol Was Reduced in Platelets from Obese Patients

**Figure 1:**
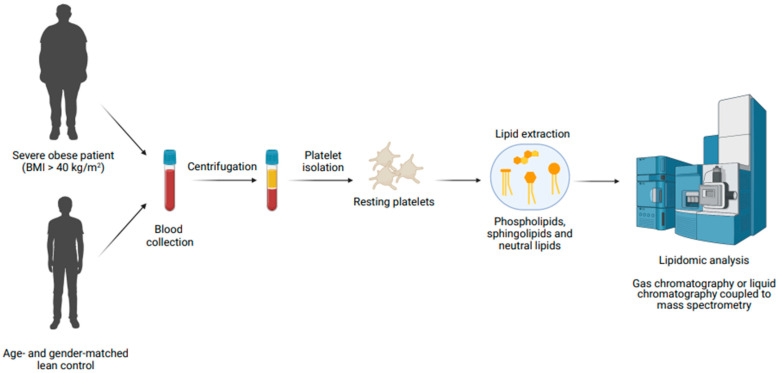
Workflow of the experimental design. The figure was created with Biorender.

Cholesterol is an important component of the plasma membrane that maintains membrane organization and fluidity over a range of physiological temperatures [12]. In the case of platelets, membranes are known to contain mostly free cholesterol and sphingomyelins (SMs) which are enriched in specialized signaling areas named lipid rafts [13]. Due to the importance of cholesterol in membrane organization, we first performed a comparative analysis of neutral lipid (i.e., cholesterol and triglyceride) content in resting washed platelets from both groups using gas chromatography (Figure 1).

**Figure 2:**
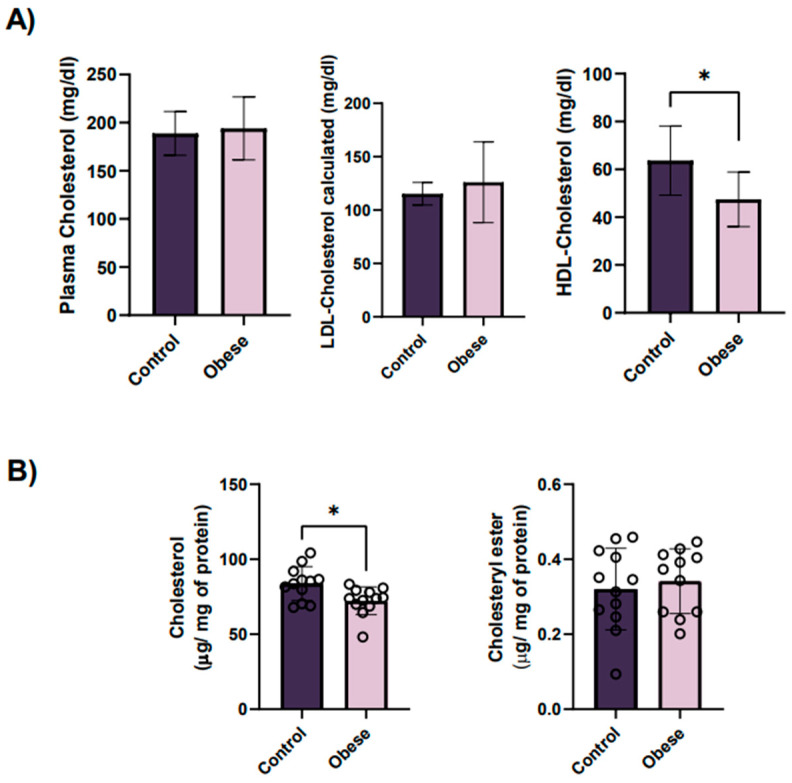
Platelet cholesterol and plasma HDL cholesterol levels are decreased in obese patients compared to lean controls, whereas no significant differences between groups exist in total plasma cholesterol levels. (**A**) Circulating cholesterol levels in blood from obese patients and their age- and gender-matched lean controls. Cholesterol levels were measured as milligrams of cholesterol per deciliter of blood. Results are represented as mean ± SD for each group (plasma cholesterol; HDL-cholesterol and LDL-cholesterol). * *p* < 0.05, according to unpaired *t*-test. (**B**) Mean cholesterol and cholesteryl ester levels in platelets from obese patients and their age- and gender-matched lean controls. Results are expressed as micrograms of lipid per milligrams of total platelet proteins and represented as mean ± SEM. * *p* < 0.05, according to unpaired *t*-test.

Even though total plasma cholesterol was found not to be different between groups (Figure 2A), with the only exception of HDL cholesterol that was lower in the obese group, the amount of total platelet cholesterol was significantly decreased in obese patients compared to controls (Figure 2B). In parallel, we did evaluate the amount of cholesteryl ester between groups. As the majority of cholesterol in platelets is free cholesterol [14], the level of cholesteryl ester was low with no differences among groups (Figure 2B).

**Figure 3:**
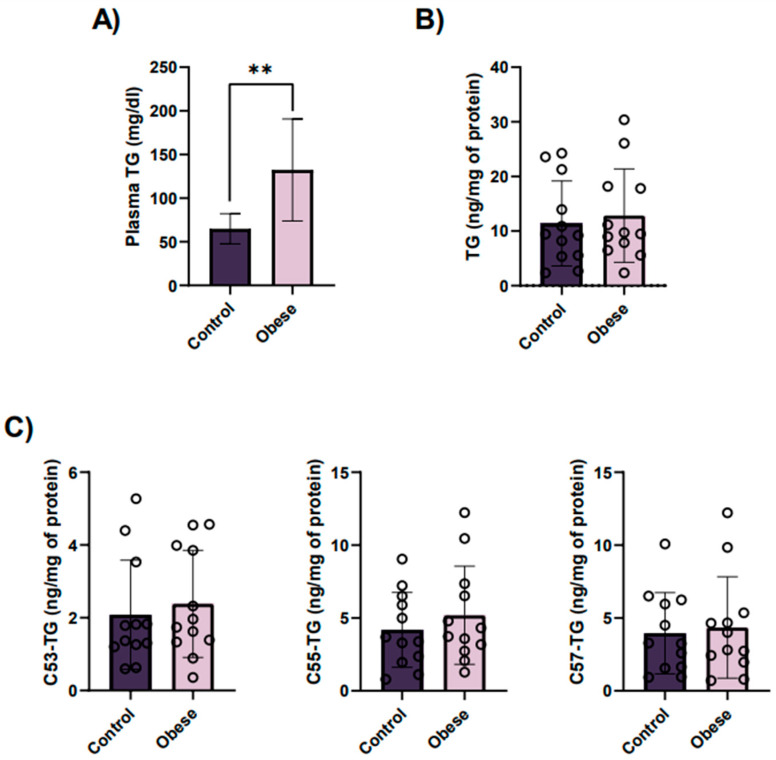
Triglyceride (TG) profile in obese patients and lean controls indicate higher plasma TG levels in the former, whereas no significant differences exist in platelet TG levels between groups. (**A**) Circulating total TG levels in blood from obese patients and their age- and gender-matched lean controls. TG levels were measured as milligrams of triglycerides per deciliter of blood. Results are represented as mean ± SD for each group. ** *p* < 0.01, according to unpaired *t*-test. Mean total TG levels (**B**) and levels of three major TG molecular species (**C**) in platelets from obese patients and their age- and gender-matched lean controls. Results are expressed as nanograms of lipids per milligrams of total platelet proteins and represented as mean ± SEM for each group.

The platelet membrane contains low amounts of other neutral lipids such as TGs. Due to the importance of the acyl chain composition in TGs, we did analyze the total content and the three main types (C53-TG, C55-TG and C57-TG). Even though we found increased levels of TGs in plasma (Figure 3A), we did not see significant differences between groups in platelets, both in the case of total TGs (Figure 3B) and main subtypes (Figure 3C).

### 2.3. Glycerophospholipids: Platelets from Obese Patients Have Reduced Levels of Phosphatidylcholine and Phosphatidylethanolamine

**Figure 4:**
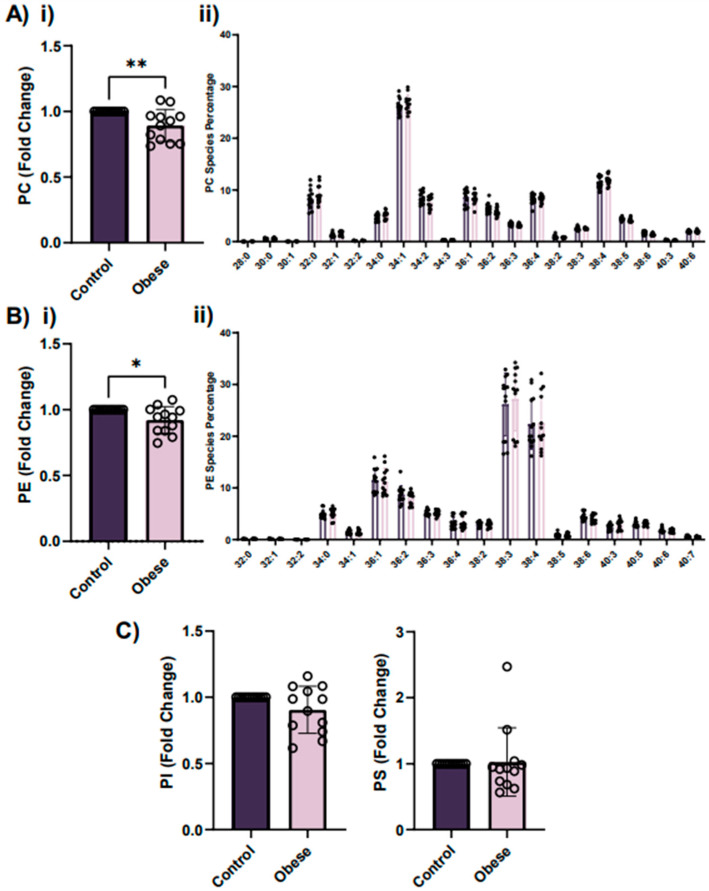
Analysis of glycerophospholipid levels in platelets from obese patients and lean controls indicates decreased levels of PC and PE in obese patients. (**A**) Levels of PC (**i**) and their molecular species (**ii**) in platelets from obese patients and their age- and gender-matched lean controls. In the case of (**i**), for each obese patient, results are expressed as fold change of area ratio (sample signal/standard signal/mg of total platelet proteins), and represented as mean fold change ± SEM for each group. Significance level is included for those cases with statistically significant differences between both groups of patients. ** *p* < 0.01, according to unpaired *t*-test. In the case of (**ii**), for 21 different molecular species of PC, results are expressed as mean percentage of the different PC molecular species ± SEM from obese patients (pink) and from age- and gender-matched lean controls (purple). (**B**) Levels of PE (**i**) and their molecular species (**ii**) in platelets from obese patients and their age- and gender-matched lean controls. In the case of (**i**), for each obese patient results are expressed as fold change of area ratio (sample signal/standard signal/mg of total platelet proteins), and represented as mean fold change ± SEM for each group. * *p* < 0.05, according to unpaired *t*-test. In the case of (**ii**), for 18 different molecular species of PE, results are expressed as mean percentage of the different PE molecular species ± SEM from obese patients (pink) and from age- and gender-matched lean controls (purple). (**C**) Levels of total PI and PS in platelets from obese patients and their age- and gender-matched lean controls. For each obese patient, results are expressed as fold change of area ratio (sample signal/standard signal/mg of total platelet proteins) and represented as mean fold change ± SEM for each group. PC, phosphatidylcholine; PE, phosphatidylethanolamine; PI, phosphatidylinositol; PS, phosphatidylserine.

Glycerophospholipids are known to be the major constituents of eukaryote cell membranes, serving as structural components and precursors for signaling and bioactive molecules [15]. In this LC-MS/MS-based lipidomics analysis, the glycerophospholipids targeted were phosphatidylcholine (PC), phosphatidylethanolamine (PE), phosphatidylinositol (PI) and phosphatidylserine (PS). According to the results, platelets from obese patients have significantly decreased PC and PE levels compared to their age- and gender-matched lean controls (Figure 4A(i),B(i)). However, no significant differences were found for PI (*p* = 0.07) and PS (*p* = 0.8) (Figure 4C).

Besides the amount of the different classes of glycerophospholipids, their molecular species composition (i.e., fatty acyl composition) is a crucial determinant for membrane physical and biological properties. The length of fatty acyl chains and the proportion of saturated and unsaturated fatty acyl such as stearic (18:0), palmitic (16:0), linoleic (18:1), arachidonic (20:4) or docosahexaenoic acids (22:6) strongly impacts membrane organization and fluidity, among others [15]. Thus, we compared the summed fatty acyl chain profiles of PC, PS, PE and PI in platelets from obese patients and their age- and gender-matched lean controls. Notably, no statistically significant differences in the profile of the different glycerophospholipid species were observed between groups (Figure 4A(ii),B(ii)) and Supplementary Figure S1).

### 2.4. Platelets from Obese Patients Have No Differences in Sphingolipid Content Compared to Lean Controls

**Figure 5:**
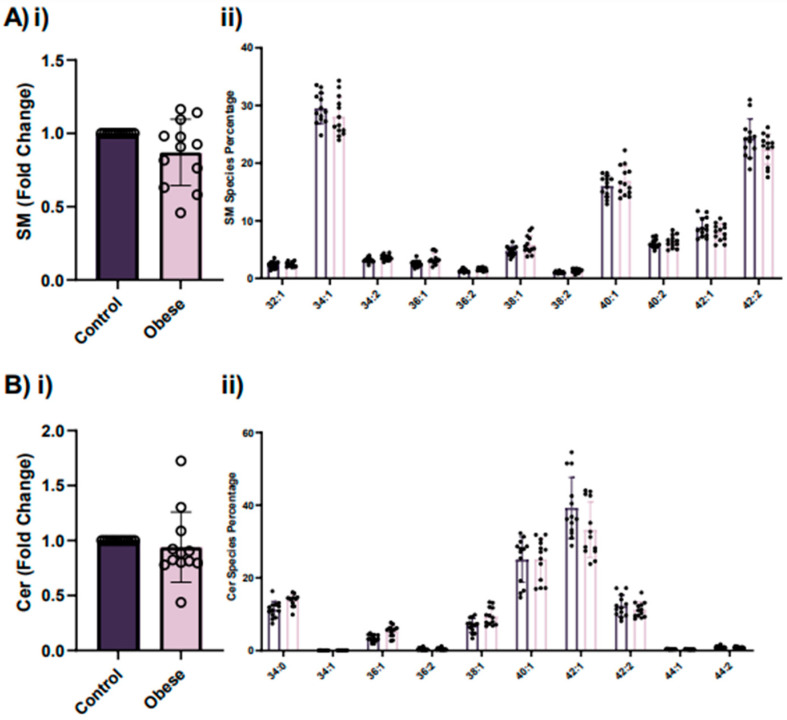
Analysis of sphingolipid levels in platelets from obese patients and lean controls shows a tendency for decreased levels of SM in the obese group. (**A**) Levels of SM (**i**) and their molecular species (**ii**) in platelets from obese patients and their age- and gender-matched lean controls. In the case of (**i**), for each obese patient, results are expressed as fold change of area ratio (sample signal/standard signal/mg of total platelet proteins) and represented as mean fold change ± SEM for each group. In the case of (**ii**), results are expressed as mean percentage of the different SM molecular species ± SEM from obese patients (pink) and from age- and gender-matched lean controls (purple). (**B**) Levels of Cers (**i**) and their molecular species (**ii**) in platelets from obese patients and their age- and gender-matched lean controls. In the case of (**i**), for each obese patient, results are expressed as fold change of area ratio (sample signal/standard signal/mg of total platelet proteins), and represented as mean fold change ± SEM for each group. In the case of (**ii**), results are expressed as mean percentage of the different Cer molecular species ± SEM from obese patients (pink) and from age- and gender-matched lean controls (purple). SM, sphingomyelin; Cers, ceramides.

Sphingolipids are a family of versatile lipids containing a backbone of sphingoid bases that play important roles in signal transduction mechanisms [16]. Lastly, the role of sphingolipids in membrane dynamics and signaling is focused on the formation and secretion of exosomes, a specific subtype of extracellular vesicles (EVs) that have been implicated in cell–cell signaling and communication [16,17]. Our research group has recently shown that circulating EVs from obese patients have a unique proteomic profile [18]. For this reason, we focused on analyzing the platelet content of sphingomyelin (SM) and ceramides (Cers). Both of them are known to be important members of the sphingolipid family. Although there were not significant differences between groups, it should be noted that the amount of sphingomyelin tended to decrease (*p* = 0.06) in the obese group (Figure 5A(i)). No significant differences were found when Cers from both groups were compared (Figure 5B(i)). Moreover, no differences were found when their molecular species were compared (Figure 5A(ii),B(ii)).

## 3. Discussion

During recent years, the lipidomics field has progressed enormously thanks to the development of high resolution and rapid scanning instruments and imaging methodologies. This has allowed researchers to explore the world of lipids in platelets and other cells. From a pathological point of view, lipids have been linked to many diseases including cancer, neurological disease, sepsis and metabolic syndromes, and their metabolism is regulated by numerous proteins [19]. Considering all the above, scientists have postulated that lipids, and their specific changes in expression, are a source of biomarkers that may be helpful for the diagnosis, follow-up and treatment of diseases.

In this study, we analyzed the lipid composition of resting platelets from a small cohort of 12 severely obese patients and their age- and gender-matched lean controls in order to identify potential lipid biomarkers of cardiovascular risk. According to the results, we found that (a) free cholesterol of platelets was reduced in platelets from obese patients; (b) TGs in plasma were increased in obese patients but no differences were observed in platelets; (c) phosphatidylcholine and phosphatidylethanolamine were significantly reduced in platelets from obese patients; and (d) sphingolipid content did not vary in platelets from both groups, although there was a tendency for a decrease in the obese group.

Lipids are essential for platelet integrity and play a fundamental role in platelet lifespan and senescence, as well as in platelet shape change, aggregation and vesicle release [13,20,21]. Consequently, the platelet lipid profile is likely dynamic, and has been reported to vary in different diseases such as liver disease [22], hyperlipoproteinemia and dyslipoproteinemias [23,24], primary defects of platelet function [25] and following dietary supplementation of n-3 polyunsaturated fat [26].

Upon activation, as it occurs during the hemostatic response, the lipid membrane composition of platelets is modified. Neutral lipids such as cholesterol are known to impact membrane fluidity and organization [15,27,28]. By interacting with fatty acyl chains, cholesterol increases membrane packing, which is important for membrane integrity and fluidity but also for the formation of lipid rafts, which are strongly involved in platelet signaling [29,30]. In the present study, we revealed that free cholesterol levels, basically located in the platelet membrane, were significantly reduced in platelets from obese patients. In line with the latter, it was recently shown that patients with arterial hypertension also presented platelet lipid alterations, including cholesterol depletion, which could lead to an alteration of platelet activation [31]. Other important lipids that play a structural role and serve as precursors of bioactive lipids during platelet activation are glycerophospholipids [32]. The activation of platelets also initiates lipid transporters to redistribute phospholipids across the membrane, allowing increased binding of coagulation factors that support the assembly of procoagulant complexes. In this context, recent platelet lipidomic quantitative studies in murine platelets, validated in human platelets, investigated main alterations in the platelet lipidome following platelet activation with major agonists, such as thrombin or collagen-related peptide (CRP) [33]. In that study, Ahrends and colleagues found that only around 20% of the platelet lipidome changed upon activation; the main alterations included sphingomyelin phosphodiesterase-1 and some glycerophospholipids [32]. Our LC-MS/MS-based targeted lipidomics approach revealed a significant decrease in the levels of both PC and PE in platelets from the severely obese patient group compared to the control group. Although in the present study we did not perform functional assays to confirm platelet hyperactivation in our obese cohort, it is important to consider that this cohort has the same characteristics as the one previously analyzed in our phosphoproteomic study published in *ATVB* in 2021 [8]. That study, in line with the present one, also pointed towards a platelet hyperactivation state in obesity, highlighting GPVI signaling as one of the most altered pathways [8].

Overall, this targeted lipidomics analysis did reveal significant decreases in free cholesterol, PC and PE in platelets from the obese compared to the age- and gender-matched control group. Remarkably, a 10% decrease in those lipids may have been due to their release from platelet membranes as extracellular vesicles (EVs), for example. The trend in PI and SM decrease in platelets from the obese group is also consistent with this hypothesis. As we mentioned, lipids are essential molecular components of EVs and elevated blood levels of platelet-derived microvesicles are frequently reported in cardiovascular diseases [33]. We recently showed that circulating EVs, mostly from platelets, in obese patients display a distinctive proteome profile with alterations in proteins from the coagulation and complement cascades. The latter correlates with the pro-inflammatory and pro-thrombotic state of the obese individuals [18]. In addition, the phospholipid and cholesterol composition of platelet-derived microvesicles generated in vitro from platelets from healthy individuals stimulated by various agonists was investigated [34]. Their phospholipid content was close to that of the plasma membrane with PC and PE being the major ones followed by sphingomyelin, PS and PI.

Our study has some limitations that should be considered, including the cohort which is limited to 12 participants per group and the sampling which was restricted to a single measurement. Some obese patients were taking antihypertensive drugs, which are known to affect platelet reactivity; however, we previously proved that such effect was balanced by the initial hypertension of those patients, thus their platelet activation levels were average with the rest of the obese group [8]. In addition, the number of patients on these chronic drugs was low and had no statistical impact on the analysis (Table 1). Related with the above, biological variation was an issue that affected the statistical analysis, making it more difficult to infer the clinical impact of the results; nevertheless, the present study paves the way for future ones with larger cohorts of patients. All control donors had a BMI below 25 Kg/m^2^, except one that had a BMI of 25.9 Kg/m^2^. This value is still way below the BMI levels of the obese group (>40 Kg/m^2^), thus the impact on the overall analysis was minor. In addition, the targeted lipidomics approach was focused on neutral lipids and major glycerophospholipids and sphingolipids; however, cells contain several thousands of lipid species. Quantitatively, minor lipids such as polyphosphoinositides, diacylglycerol and eicosanoids are known to play critical roles in platelet signaling and function, but they were not investigated in this instance.

Even though platelets have been thoroughly investigated, our knowledge of their biology continues to expand with lipidomic characterization. Combining this level of analytical ability with the study of large cohort sample sets, a new generation of MS technology has the potential to transform our understanding of disease mechanisms. This is the first study describing a comparative analysis of the platelet lipid composition of severely obese patients and their age- and gender-matched lean controls. It provides novel information on the status of platelet lipids in obesity, highlights the importance of studying the generation of platelet-derived extracellular vesicles in obese patients and may guide further studies to obtain new mechanistic insights into the behavior of platelets in this multifactorial disease.

## 4. Materials and Methods

### 4.1. Chemicals and Reagents

All reagents were purchased from Sigma-Aldrich (Saint Louis, MO, USA) unless specified. The liquid chromatography solvent was HPLC-grade and purchased from Acros Organics. Synthetic lipid standards (Cer d18:1/18:0, Cer d18:1/15:0, PE 12:0/12:0, PE 16:0/16:0, PC 13:0/13:0, PC 16:0/16:0, SM d18:1/18:0, SM d18:1/12:0, stigmasterol, cholesteryl heptadecanoate and glyceryl trinonadecanoate) were purchased from Avanti Polar Lipids and Sigma-Aldrich.

### 4.2. Patients and Study Design 

The experimental design is described in Figure 1. Twelve severely obese patients (BMI > 40 kg/m^2^) who had been referred by their general practitioner to an outpatient clinic in the Endocrinology Unit at the Santiago de Compostela University Hospital (Spain) participated in the study. The study was approved by the local Ethics Committee (Galician Investigation Ethics Committee; Code No. 2009/270; date: 22 December 2015) and developed according to the principles outlined in the Declaration of Helsinki. Written informed consent was obtained from all participants prior to blood extraction. None of the severely obese patients were diabetic or had other major comorbidities. Exclusion criteria included coagulation or platelet-associated disorders, and having taken antiplatelet drugs 10 days prior to blood donation. The age- and gender-matched control group was formed by 12 healthy normal weight volunteers recruited at the Santiago de Compostela University Health Service.

### 4.3. Blood Sampling and Platelet Preparation

Blood samples were collected from obese and lean-matched controls in 3.2% (*w*/*v*) trisodium citrate tubes (Vacuette^®^) and processed within 60 min. Washed platelets were isolated as previously described by a method established to limit contamination from other blood cells and plasma proteins [35]. Washed platelets were resuspended in modified Tyrode buffer (134 mmol/L NaCl, 0.34 mmol/L Na_2_HPO_4_, 2.9 mmol/LKCl, 12 mmol/L NaHCO_3_, 20 mmol/L HEPES, 5 mmol/L glucose, 1 mmol/L MgCl_2_, pH 7.3) at the desired concentration (8 × 10^8^ platelets/mL) and allowed to rest for 30 min at room temperature. After that, they were incubated with 1 mM of EGTA for 5 min to prevent activation during centrifugation and centrifuged at 1000× *g* for 10 min. Platelet pellets were snap frozen in liquid nitrogen and immediately stored at −80 °C until the lipidomic analysis.

### 4.4. Lipid Extraction

Platelet pellets (2 × 10^8^ platelets per pellet) were resuspended in 1 mL of methanol/water 5 mM EGTA (2:1, *v*/*v*) by sonication using a probe sonicator (130 W, 7 s, output 30%). Fifty microliters of the homogenate were used for protein quantification after methanol evaporation. Lipids were extracted according to a modified Bligh and Dyer [12] method in dichloromethane/methanol (2% acetic acid)/water (2.5:2.5:2, *v*/*v*/*v*), in the presence of the internal standards (Cer 18:1/12:0 16 ng; PE 12:0/12:0 180 ng; PC 13:0/13:0 16 ng; SM 18:1/12:0 16 ng; PI 14:1/17:0 30 ng; PS 12:0/12:0 156.25 ng; 4 µg of stigmasterol, 4 µg of cholesteryl heptadecanoate and 16 µg of glyceryl trinonadecanoate). After vigorous mixing and centrifugation at 2500 rpm for 6 min, the organic phase was collected and dried under a stream of N_2_. The samples were then resuspended in 50 µL of methanol and stored at −20 °C prior to quantification of glycerophospholipids, ceramide and sphingolipids. Moreover, 10 µL of the samples were dried under a stream of N_2_ and resuspended in 20 µL of ethyl acetate to quantify neutral lipids.

### 4.5. Neutral Lipid Analysis

Neutral lipid profiling was performed using gas chromatography on a FOCUS Thermo electron system using a Zebron-1 Phenomenex fused-silica capillary column (5 m × 0.32 mm i.d, 0.50 mm film thickness) [36]. Oven temperature was programmed from 200 to 350 °C at a rate of 5 °C per min and the carrier gas was hydrogen (0.5 bar). The injector and the detector were at 315 °C and 345 °C, respectively.

### 4.6. Glycerophospholipid and Sphingolipid Profiling

Glycerophospholipid and sphingolipid profiling was performed using an Agilent 1290 UPLC system coupled to a G6460 triple quadrupole spectrometer (Agilent Technologies, Santa Clara, CA, USA). A Kinetex HILIC column (Phenomenex, 50 × 4.6 mm, 2.6 µm) was used for liquid chromatography (LC) separations where the flow rate of the mobile phase was 0.3 mL/min and the injection volume was 5 µL. The column temperature was controlled at 40 °C. The mobile phase (A) was acetonitrile and (B) was 10 mM ammonium formate in water at pH 3.2. The gradient was as follows: from 10% to 30% B in 10 min; 100% B for 10–12 min and then back to 10% B at 13 min for 1 min re-equilibrium prior to the next injection. An electrospray source was used in positive (for Cer, PE, PC and SM analysis) and negative ion mode (for PI and PS analysis). The collision gas was nitrogen. Needle voltage was set at +4000 V. Several scan modes were used. First, to obtain the naturally different species’ mass, we analyzed cell lipid extracts with a precursor ion scan of 184 m/z, 241 m/z and 264 m/z for PC/SM, PI and Cer, respectively; and a neutral loss scan of 141 and 87 for PE and PS, respectively. The collision energy optimums for Cer, PE, PC, SM, PI and PS were 25 eV, 20 eV, 30 eV, 25 eV, 45 eV and 22 eV, respectively. Then, the corresponding SRM transitions were used to quantify different PL species for each class. Two MRM acquisitions were necessary because of important differences between PL classes. Data were treated using QqQ Quantitative (vB.05.00) and Qualitative analysis software (vB.04.00).

### 4.7. Statistical Analysis

Categorical variables from obese patients and lean-matched controls are expressed as percentages and were compared using Fisher’s exact test. Continuous variables are expressed as mean ± SD. Normal distribution was tested using the Shapiro–Wilk test; those normal-distributed variables were compared using the Mann–Whitney test. In all cases, values of *p* < 0.05 were considered statistically significant.

## Figures and Tables

**Table 1:** Main clinical and physiological characteristics of obese patients and lean controls.

	Control (*n* = 12)		Obese (*n* = 12)		
	Value (Mean ± SD)	*n*	Value (Mean ± SD)	*n*	*p*-value
Females (%)	75.00%	12	75.00%	12	-
Age (Years)	41.75 ± 12.34	12	42.50 ± 11.47	12	0.8789
BMI (kg/m^2^) ****	23.02 ± 1.91	12	45.89 ± 5.21	12	<0.0001
Diabetics (%)	0%	12	0%	12	>0.9999
Laboratory measurements					
Hemoglobin (g/dL)	13.67 ± 1.28	10	14.09 ± 1.40	12	0.4730
Leukocytes ×10^3^/µL **	5.51 ± 1.37	10	7.29 ± 1.54	12	0.0099
Platelets ×10^3^/µL *	222.56 ± 58.98	10	272.40 ± 44.01	12	0.0385
Mean Platelet Volume (fL)	8.84 ± 1.11	10	9.07 ± 1.08	12	0.6347
Glucose (mg/dL) ***	79.00 ± 4.37	10	90.08 ± 6.79	12	0.0002
Creatinin (mg/dL)	0.72 ± 0.13	10	0.76 ± 0.18	12	0.5084
Cholesterol (mg/dL)	188.90 ± 22.72	10	194.00 ± 32.71	12	0.6820
Triglycerides (mg/dL) **	64.88 ± 17.32	8	132.30 ± 58.39	12	0.0056
Drug treatment					
Tyrode drugs	0%	12	8.33%	12	>0.9999
Anti-cholesterol drugs	0%	12	8.33%	12	>0.9999
ACE drugs	0%	12	16.67%	12	0.4783
Ca^2+^ channel blockers	0%	12	8.33%	12	>0.9999
Skeletal muscle relaxants	0%	12	8.33%	12	>0.9999

BMI: body mass index, ACE: angiotensin-converting enzyme. Data are expressed as mean ± SD. * *p* < 0.05, ** *p* < 0.01, *** *p* < 0.001, **** *p* < 0.0001.

## Supplementary Materials

The following supporting information can be downloaded at: https://www.mdpi.com/article/10.3390/ijms23158326/s1.
