# Supplementary material for: Platelet Lipidome Fingerprint: New Assistance to Characterize Platelet Dysfunction in Obesity
Source: Int J Mol Sci. 2022 Jul 28;23(15):8326. doi: 10.3390/ijms23158326 (PMC9369067; doi:10.3390/ijms23158326)
Supplement: Supplementary file 1 [file ijms-23-08326-s001.zip › ijms-1819018-supplementary.pdf]

Platelet lipidome fingerprint: new assistance to characterize platelet dysfunction in obesity

Gaëtan Chicanne[1*], Maria N. Barrachina[2,3*], Anaelle Durbec[1,4], Justine Bertrand-Michel[1,4], Sara Troitiño[2], Lidia Hermida-Nogueira[2], Aurelio M. Sueiro[5], María Pardo[6], Bernard Payrastre[#1,7] and Ángel García[#2].

[1] Institute of Metabolic and Cardiovascular Disease, Inserm UMR1297, University of Toulouse 3, 31024 Toulouse, France.

[2] Platelet Proteomics Group, Center for Research in Molecular Medicine and Chronic Diseases (CIMUS), Universidade de Santiago de Compostela, and Instituto de Investigación Sanitaria de Santiago (IDIS), Santiago de Compostela, Spain.

[3] Vascular Biology Program, Boston Children's Hospital, Harvard Medical School, Boston, USA.

[4] MetaboHUB-MetaToul, National Infrastructure of Metabolomics and Fluxomics, Toulouse, 31077, France.

[5]Grupo de Endocrinología Molecular y Celular, Instituto de Investigación Sanitaria de Santiago (IDIS), Servicio de Endocrinología, Xerencia de Xestión Integrada de Santiago (XXS), Santiago de Compostela, Spain.

[6] Grupo Obesidómica, Instituto de Investigación Sanitaria de Santiago (IDIS), Xerencia de Xestión Integrada de Santiago, Santiago de Compostela, Spain.

[7] Laboratory of Haematology, University Hospital of Toulouse, 31059 Toulouse, France.

* Co-authors

# Co-senior authors

SUPPLEMENTARY FIGURES

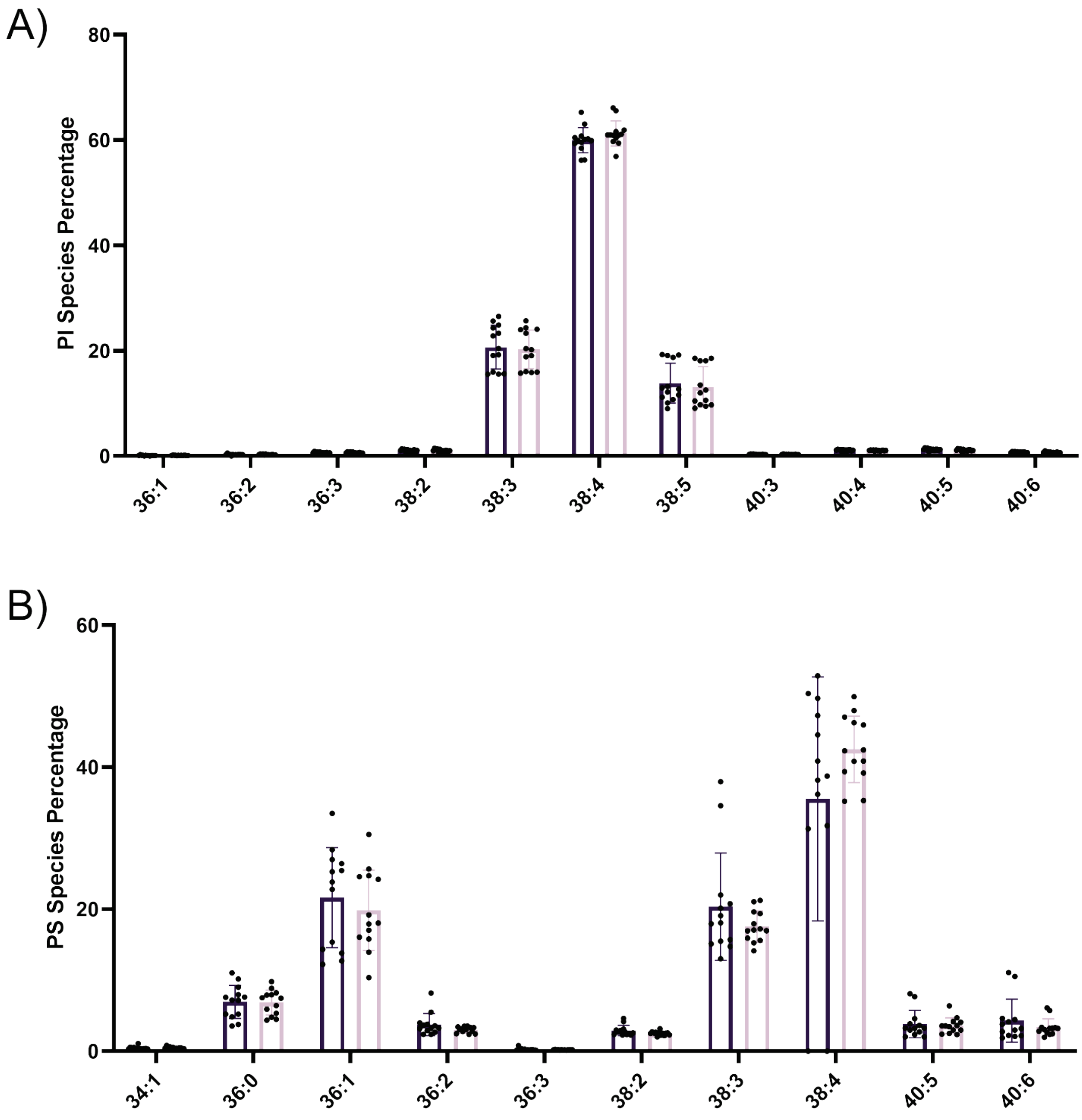

Supplementary Figure S1. PI and PS molecular species levels in platelets from obese patients and lean controls. A) Levels of 11 different molecular species of PI from obese patients and their age- and gender-matched lean controls. Results are expressed as mean percentage of the different PI molecular species ± SEM from obese patients (pink) and from age- and gender-matched lean controls (purple). **B)** Levels of 10 different molecular species of PS from obese patients and their age- and

gender-matched lean controls. Results are expressed as mean percentage of the different PS molecular species ± SEM from obese patients (pink) and from age- and gender-matched lean controls (purple). SM, sphingomyelin; Cer, ceramides.

Supplementary Table S1. Expanded clinical and physiological characteristics of obese patients and lean controls.

	CONTROL (n=12)		OBESE (n=12)	
	Value (Mean ± SD)	*n*	Value (Mean ± SD)	*n*
**Females (%)**	75.00%	12	75.00%	12
**Age (Years)**	41.75 ± 12.34	12	42.50 ± 11.47	12
**BMI (kg/m^2) ******	23.02 ± 1.91	12	45.89 ± 5.21	12
**Diabetics (%)**	0%	12	0%	12
**Laboratory measurements**				
**Hemoglobin (g/dl)**	13.67 ± 1.28	10	14.09 ± 1.40	12
**HDW, Hemoglobin Distribution Width (%) ***	2.30 ± 0.13	7	2.56 ± 0.31	12
**Leukocytes x10^3/µL ****	5.51 ± 1.37	10	7.29 ± 1.54	12
**Platelets x10^3/µL ***	222.56 ± 58.98	10	272.40 ± 44.01	12
**Mean Platelet Volume (fL)**	8.84 ± 1.11	10	9.07 ± 1.08	12
**PDW, Platelet Distribution Width (%)**	49.63 ± 9.43	7	48.65 ± 9.18	12
**Glucose (mg/dl) *****	79.00 ± 4.37	10	90.08 ± 6.79	12
**Creatinin (mg/dl)**	0.72 ± 0.13	10	0.76 ± 0.18	12
**Albumin (mg/dl)**	4.39 ± 0.17	7	4.26 ± 0.21	11
**Proteins (mg/dl)**	6.77 ± 0.33	6	6.86 ± 0.41	11
**Cholesterol (mg/dl)**	188.90 ± 22.72	10	194.00 ± 32.71	12
**LDL-Cholesterol calculated (mg/dl)**	115.33 ± 10.60	6	126.10 ± 37.85	11
**No HDL-Cholesterol (mg/dl)**	129.00 ± 11.88	6	138.90 ± 33.85	11
**HDL-Cholesterol (mg/dl) ***	63.67 ± 14.47	6	47.45 ± 11.42	11
**Triglycerides (mg/dl) ****	64.88 ± 17.32	8	132.30 ± 58.39	12
**Drug treatment**				
**Tyrode drugs**	0%	12	8.33%	12
**Anti-cholesterol drugs**	0%	12	8.33%	12
**ACE drugs**	0%	12	16.67%	12
**Ca^{2+} channel blockers**	0%	12	8.33%	12
**Skeletal muscle relaxants**	0%	12	8.33%	12

BMI: Body Mass Index, ACE: angiotensin-converting enzyme. Data are expressed as mean ± SD.

* $p<0.05$, ** $p<0.01$, *** $p<0.001$, **** $p<0.0001$.
